# Alcohol Modulation of Amyloid Precursor Protein in Alzheimer’s Disease

**Published:** 2020-07

**Authors:** Steven A. Masi, Madhavan P. Nair, Michael Vigorito, Tinchun Chu, Sulie L. Chang

**Affiliations:** 1 Institute of Neuroimmune Pharmacology, Seton Hall University, South Orange, USA; 2 Immunology and Nano-Medicine, Florida International University, Miami, USA; 3 Department of Psychology, Seton Hall University, South Orange, USA.; 4 Department of Biological Sciences, Seton Hall University, South Orange, USA

**Keywords:** Alcohol consumption, Alzheimer’s Disease, Ingenuity Pathway Analysis, Amyloid precursor protein, Inflammatory response

## Abstract

Heavy alcohol use increases the risk of Alzheimer’s Disease (AD); however, the underlying mechanisms are not addressed. The key chemical of alcohol beverages is ethanol (EtOH), and acetaldehyde is its key toxic metabolite. QIAGEN Ingenuity Pathway Analysis (IPA) bioinformatics tool was used to investigate and compare the holistic impact of EtOH and acetaldehyde on AD. An extensively researched biomarker of AD pathologies is amyloid-beta of which the precursor is amyloid precursor protein (APP). Molecules associated with APP or EtOH were collected from the QIAGEN Knowledge Base, and 313 molecules were overlapping between the molecule sets. Using the “Pathway Explorer” tool, 40 of the 313 molecules were found to change due to EtOH exposure and influence APP and were compared with acetaldehyde-mediated molecule expression changes. A pathway analysis of the findings related to these 40 molecules showed that EtOH increases APP expression at a confidence of *p* = 0.056 (z-score = 1.91, two-tailed). Among the top 10 IPA canonical pathways, ranked by the Benjamini-Hochberg corrected Fisher’s Exact Test, identified through the “Core Expression Analysis” feature of IPA, revealed that neuroinflammation was associated at the highest confidence (*p*=5.97E-73). Our study suggests involvement of the neuroinflammation pathway in alcohol modulation of APP as a potential causal factor in AD.

## 1. Introduction

Alzheimer’s Disease (AD) is a complex neurodegenerative disorder marked by progressive buildup of β amyloid plaques, neurofibrillary tangles, neuroinflammation, and neuronal injury and loss. The influential “amyloid cascade hypothesis” implicated the buildup of insoluble extracellular amyloid beta (Aβ) protein deposits in the form of amyloid fibrils as the principal causal mechanism of AD pathogenesis [1–5]. Although supported by early studies the hypothesis has proven problematic over the last two decades [6,7]. Nevertheless, Aβ accumulation as a causal mechanism of AD remains an active area of investigation. The Aβ monomer, cleaved from the amyloid precursor protein (APP), aggregates not only into the amyloid fibrils that is the hallmark of the amyloid cascade hypothesis but also into other assemblies that have been implicated in AD such as protofibrils and oligomers [8]. Moreover, although APP is expressed ubiquitously and is implicated in brain development and adult brain plasticity, increased expression of APP is associated with AD [9–11].

The amyloid cascade hypothesis also stimulated investigations of the overexpression of APP in transgenic cellular and animal models of AD [10]. Most of these models reflect the early onset familial form of AD associated with mutations in the APP gene or the presenilin genes of the γ-secretase complex. However, familial AD is uncommon relative to the late-onset sporadic form of the disorder, the latter making up approximately 90% of AD cases [12, 13]. In contrast to the familial form, sporadic AD is not inherited in an autosomal dominant fashion but is associated with the inheritance of a variant of the apolipoprotein E (ApoE) gene. The risk of sporadic AD rises with increasing frequency of the ApoE4 variant. Moreover, while amyloid fibrils are insoluble and form the tangles characteristic of AD, the Aβ oligomers are soluble and possibly spread throughout the brain [8]. Carriers of the ApoE4 allele show decreased Aβ clearance from extracellular space, leading to gradual accumulation of Aβ over time [10]. Thus, a major pathogenic pathway in sporadic AD is the inefficient clearance of soluble Aβ [14]. Soluble Aβ has also been implicated in microglia-mediated pro-inflammatory activation [15]. Indeed, increasing evidence suggests that the risk of AD is strongly associated with factors that induce long-lasting neuroinflammation [15–18].

A link between systemic inflammation and neurodegeneration has also been receiving increasing attention [19–22]. For example, lipopolysaccharide (LPS) induces enhancement of Aβ generation and concomitant cognitive impairment in mice [23]. In an APP mouse model low-dose LPS treatment exacerbated β-amyloidosis [24]. In human AD patients, systemic infection hastens cognitive decline [20]. Evidence that endotoxins contribute to several neurodegenerative disorders is also emerging [25]. Targeting LPS, TLR4/CD14 receptors and Gram-negative bacteria has also been proposed as a new emerging strategy to slow or prevent sporadic AD [26]. The finding that systemic inflammation induced by peripheral disease causes neuroinflammation broadens the nongenetic or epigenetic risk factors that contribute to AD including alcohol abuse and addiction.

There is a body of literature indicating that exposure to moderate amounts of alcohol consumption is associated with a reduced risk of dementia [27, 28] and results in neuroprotective effects against AD-associated pathology [29]. Although the protective effects of moderate levels of alcohol intake continue to be controversial, longitudinal studies have linked heavy alcohol consumption with as much as a 3-fold increase in the risk of developing AD [1–5]. Systemic inflammatory changes in alcoholics is well-established [30]. Chronic alcohol consumption has been shown to increase systemic inflammation by inducing intestinal hyperpermeability (leaky gut) through the disruption of epithelial cells leading to increased transmission of pathogens and toxic substances into the bloodstream. A leaky gut leads to increased endotoxin concentrations (i.e. LPS) and associated systemic inflammation [31–36]. Alcohol abuse may also cause neuroinflammation by interfering with normal gut microbiota [37] or disruption of the anti-inflammatory activity of the inflammatory reflex of the vagus nerve and gut metabolic homeostasis [37, 38]. Thus, we postulate that ethanol (EtOH) contributes to AD pathology by inducing neuroinflammation through the modulation of APP expression. Furthermore, although there are mechanisms of action that are directly influenced by EtOH, many *in vivo* and *in vitro* studies have suggested that one of the primary drivers of the deleterious effects of alcohol come from liver processing EtOH into the toxic metabolite, acetaldehyde [39, 40]. If this hypothesis is correct there should be an association between molecules that change during EtOH exposure and molecules that influence APP in the context of well-characterized cell signaling and metabolic pathways (i.e. canonical pathways). Furthermore, if acetaldehyde fully mediates the biological influence of EtOH in this context, acetaldehyde associated molecules should account for all of the molecule changes that influence APP.

Various bioinformatics tools are effective in data mining applications. We are a licensed user of the QIAGEN Ingenuity Pathway Analysis (IPA) Analysis Match CL. The QIAGEN Knowledge Base (QKB) is a repository of curated information created from over seven million individually modeled relationships between diseases, drugs, biological entities (e.g., genes, proteins, and metabolites) and processes, including the type of process (e.g., expression, molecular cleavage, phosphorylation, etc.) and the published results of omics experiments (e.g., increased or decreased expression). The information is extracted primarily from the full text of journal articles from the scientific literatures and manually curated by research experts. In this study, we employed several QIAGEN IPA bioinformatics data mining tools (QIAGEN Inc., https://www.qiagenbioinformatics.com/products/ingenuitypathway-analysis) to test our hypothesis by 1) identifying the EtOH- and APP-associated molecules found in the QKB; 2) characterizing the influence of EtOH exposure on the common molecules associated with EtOH and APP; and 3) evaluating the molecules identified with both EtOH and APP using the known canonical pathways, and using the findings of a canonical pathway analysis for APP and fertility as negative control data because APP and fertility have no a-priori relationship.

## 2. Methods and Resources

### 2.1. Ingenuity Pathway Analysis Software

**Figure 1:**
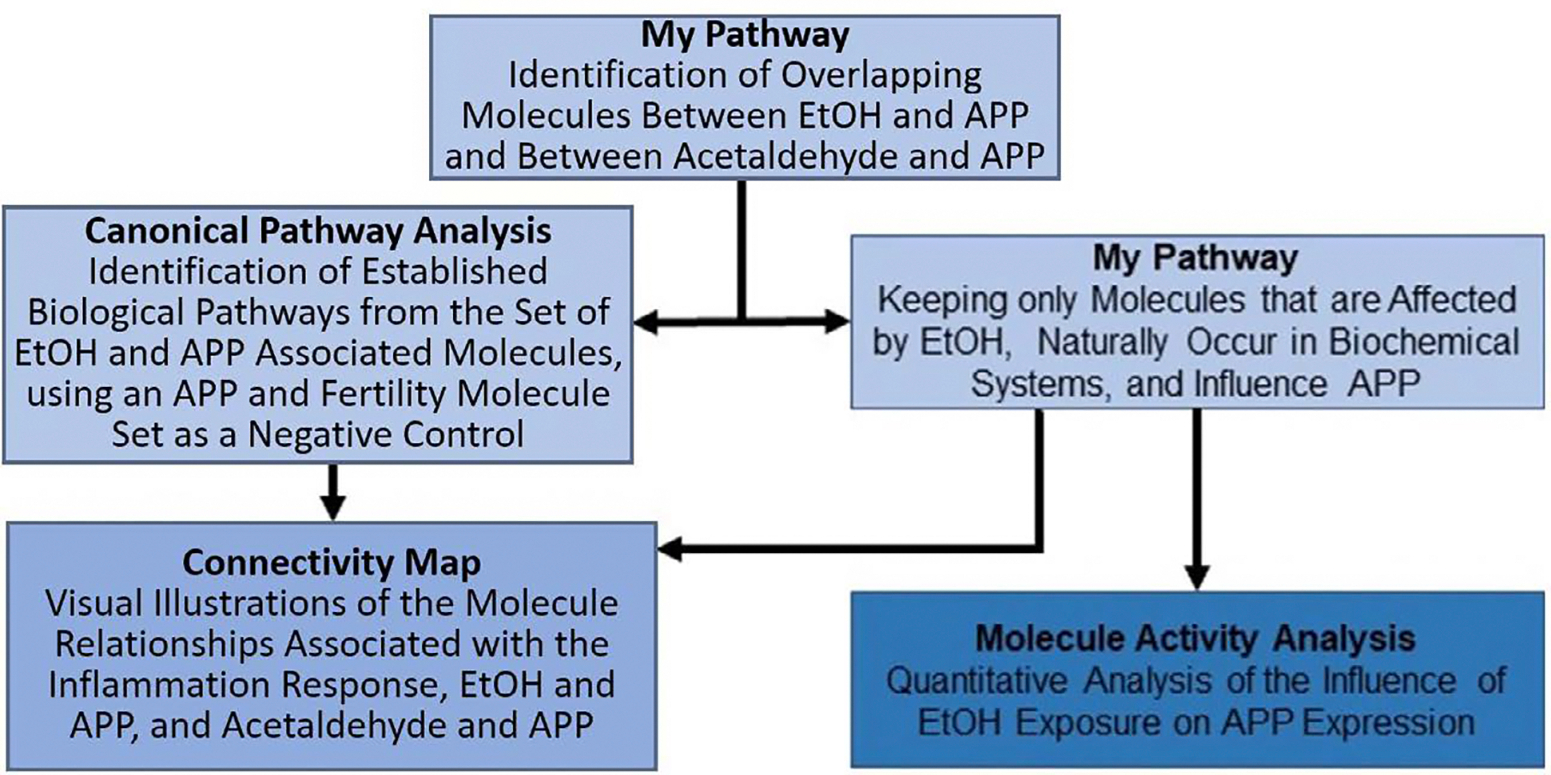
The flow of information from bioinformatics, data mining tools used to analyze the relationship between EtOH exposure and APP. Using the My Pathway tool, molecules that have been identified to be associated with EtOH exposure or with acetaldehyde (a toxic metabolite of EtOH) were compared with the molecules associated with APP. A canonical pathway analysis was then performed on the set of EtOH associated molecules to identify the most relevant biological pathways associated with the overlap of APP and EtOH. The My Pathway tool was further used to keep only those molecules of the EtOH molecule set that were specifically affected by EtOH, that naturally occur in biochemical systems, and that have been shown to influence APP. The Connectivity Map tool was used to visually illustrate the relationships between EtOH and APP, acetaldehyde and APP, and identify the molecules of the refined EtOH and APP molecule set associated with inflammatory response. Finally, a quantitative confidence calculation was performed on the biological relationship findings of the EtOH and APP molecule subset to produce the likelihood of a change in APP expression following EtOH exposure.

The IPA Analysis Match CL license was purchased from QIAGEN for using all features and tools of the IPA software. All data used for the EtOH and APP analysis for this study were retrieved from the QIAGEN Knowledge Base (QKB) on March 16, 2020 and all data used for the acetaldehyde and APP analysis, and APP and fertility analysis for this study were retrieved from the QKB on July 4, 2020. The IPA tools were used to analyze data, findings and datasets with respect to the known metabolic and signaling pathways (QIAGEN Inc., https://www.qiagenbioinformatics.com/products (Figure 1).

### 2.2. My Pathway: Molecule Relationship Mapping

IPA’s “My Pathway” feature is to identify and organize molecule and biological relationship information from published research data from *in vitro* and *in vivo* research of human, rat, and mice subjects from a variety of biological contexts stored in the QKB. The “My Pathway” tool was used to identify the relationship information of biologically relevant molecules associated with EtOH and APP and with acetaldehyde and APP. These molecules were used to reveal the common pathways between EtOH and APP and between acetaldehyde and APP, respectively, and identify the molecular processes modulated by EtOH exposure that influence APP expression.

#### 2.2.1. Identification of overlapping molecules

The “Grow” and “Pathway Explorer” tools were used to add biological context to the molecules shared by EtOH and APP, as well as, the molecules shared by acetaldehyde and APP. For both the holistic EtOH analysis and the acetaldehyde analysis, “Connect,” “Trim,” and “Keep” tools were used to keep only molecules relevant to EtOH and APP and relevant to acetaldehyde and APP respectively. Furthermore, these tools were used to identify the subset of molecules that had relevance in quantifying the change in APP expression following EtOH exposure in a typical biological system. This process involved removing all molecules that were not naturally occurring in biological systems (e.g., chemical drugs or toxicants), removing the molecules that did not have known changes to EtOH exposure, and removing the molecules that did not have a known influence on APP.

#### 2.2.2. Connectivity mapping

Molecule relationships added with the “Grow” and “Pathway Explorer” tools were analyzed by employing the “Molecule Activity Predictor (MAP)” tool to simulate the change in activity processes (i.e., expression, transcription, activation, inhibition or phosphorylation) in response to the exposure or expression of other molecules. A visual illustration of the relationships based on the findings stored in the QKB was then produced. The MAP tool was used to illustrate the holistic influence of EtOH exposure on APP and the Molecules identified as being associated with EtOH and APP. The MAP tool was used with the findings underlying the known relationships between EtOH and APP to show the impact of EtOH exposure on molecules mediating the interaction between EtOH and APP, and the change in those intermediary molecules caused by EtOH exposure that have been shown to influence APP. In addition to this holistic analysis of EtOH exposure on APP expression, the specific influence of the EtOH metabolite acetaldehyde on APP expression was identified using the same “Grow,” “Pathway Explorer,” and MAP tools to reveal the molecules associated with both acetaldehyde and APP using the data available in the QKB on July 4, 2020.

### 2.3. Canonical Pathway Analysis

The ”Canonical Pathway Analysis” of the “Core Analysis, Expression Analysis” function of IPA was implemented to analyze molecule datasets by finding overlapping molecules between the datasets and the molecules of known metabolic and signaling pathways. The dataset was then assigned a significance value for each of the 705 canonical pathways stored in the QKB using a Benjamini-Hochberg Corrected Fisher’s Exact Test to obtain a right-tailed *p* value, showing the likelihood of finding that number of overlapping molecules from the dataset in the canonical pathway. The “Canonical Pathway Analysis” was used to identify the pathways associated with the set of molecules that have relationships with both EtOH and APP. This allowed for the identification of biological signaling pathways that are involved in the modulation of APP expression due to EtOH exposure, as well as the common systems that are influenced by EtOH and APP. Furthermore, a canonical pathway analysis was performed on APP and fertility that has no a-prior association with APP, as a negative control to confirm that the observed relationship between EtOH and APP is a specific one and does not reflect non-specific biological processes associated with disease in general.

### 2.4 Quantitative Analysis of the Influence of EtOH Exposure on APP

The “Downstream Effect Analysis” algorithm as described by Krämer (2014) [41] is used to calculate a confidence value for the relationship change in activity (i.e., expression, transcription, activation, inhibition or phosphorylation) based on the number of findings and consistency of the findings supporting that change stored in the QKB. With randomized molecule relationships, the distribution of predicted changes approaches a normal distribution, such that the aggregation of weights together generates a “local z-score” which corresponds to a z-statistic of a normal distribution and a confidence measure that a finding of such a consistency would be found given a random set of molecules. This algorithm was used to confirm a confidence for the change in APP expression in response to EtOH exposure, based on the changes following EtOH exposure of molecular processes that are known to influence APP. Findings pulled from 410 references as data points were used to simulate the impact of EtOH on the identified intermediary molecules between EtOH and APP and to simulate the impact of the simulated change in the intermediary molecules on APP expression. This analysis was used to calculate the direction of the change in APP in response to EtOH exposure and the likelihood that the results would have been found given a random normal distribution of finding data points.

## 3. Results

### 3.1 Molecules associated with EtOH and APP

**Figure 2:**
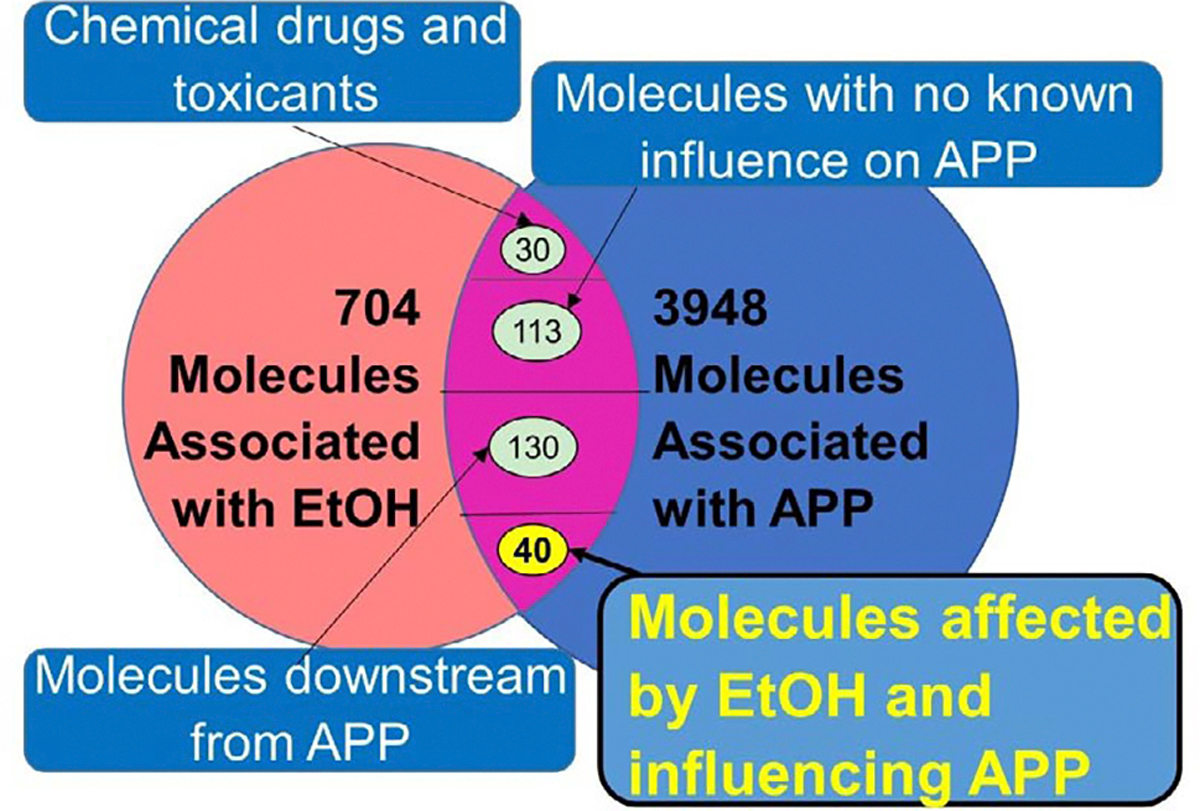
Categorization of the 313 overlapping molecules associated with EtOH and APP, to identify the molecules that could be used for the algorithmic analysis of the impact of EtOH exposure on APP Expression.

Using IPA’s “Grow” tool, 704 molecules were identified to be associated with EtOH from the QKB. APP was found to have 3948 molecules associated with it. The EtOH- and APP-associated molecule sets were found to have 313 overlapping molecules (Figure 2).

### 3.2 Molecules Affecting APP Expression in a Typical Biological System Following EtOH Exposure

From the 313 molecules that were associated with both APP and EtOH, 130 molecules were removed for being downstream from APP, 30 molecules were removed for not naturally occurring in biological systems (i.e., chemical drugs and toxicants), and 113 molecules were removed for not having a known change from EtOH exposure or a known influence on APP. This resulted in 40 molecules that naturally occur in a biological system and had a known response to EtOH exposure and a known influence on APP (Figure 2).

### 3.3 The Influence of EtOH Exposure on APP Expression

**Figure 3:**
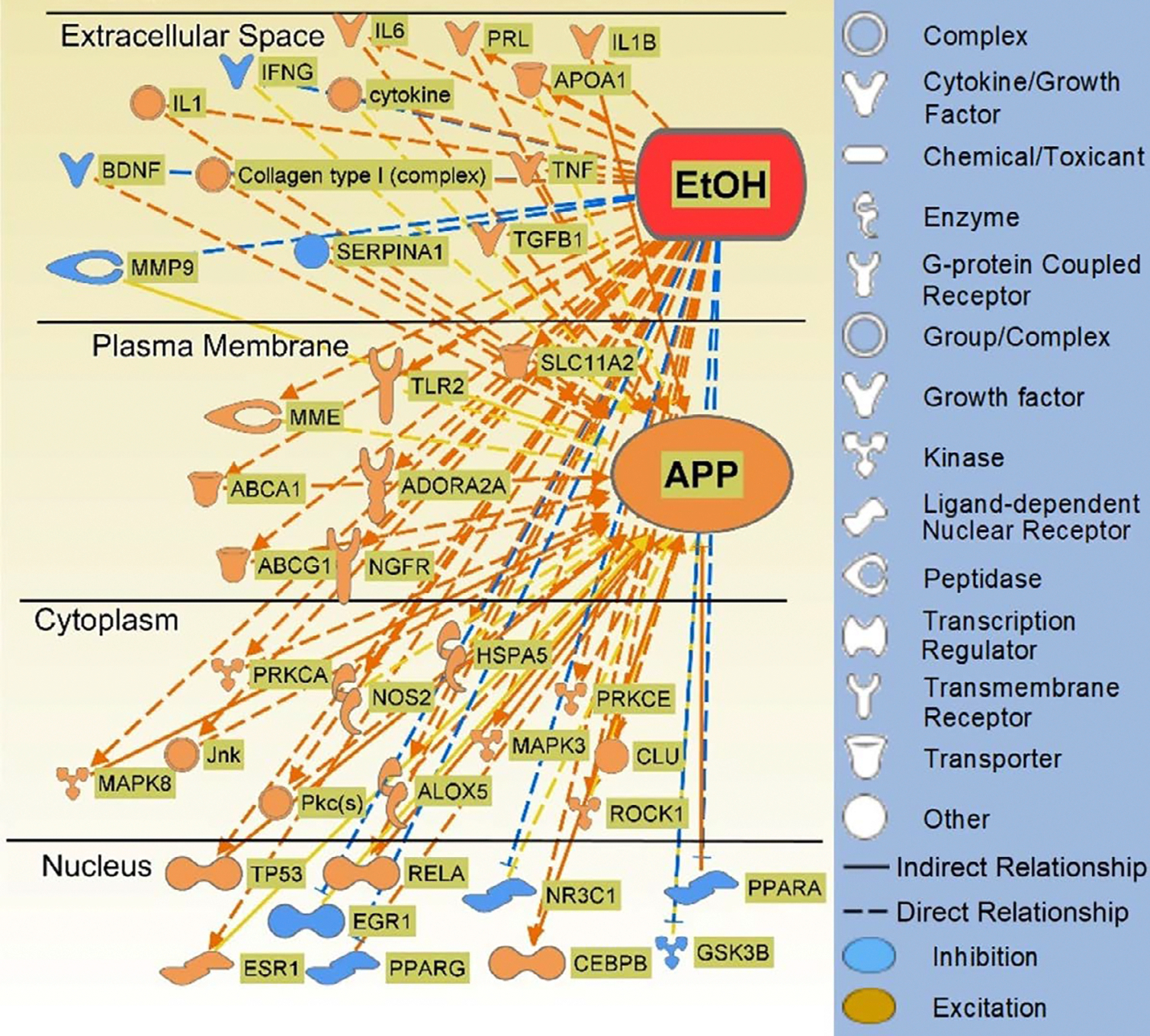
Connectivity Map of the direct (solid lines) and indirect (dotted lines) molecule relationships of the 40 molecules which are influenced by EtOH exposure and which influence APP. For the full list of 40 molecules see Table 1.

**Figure 4:**
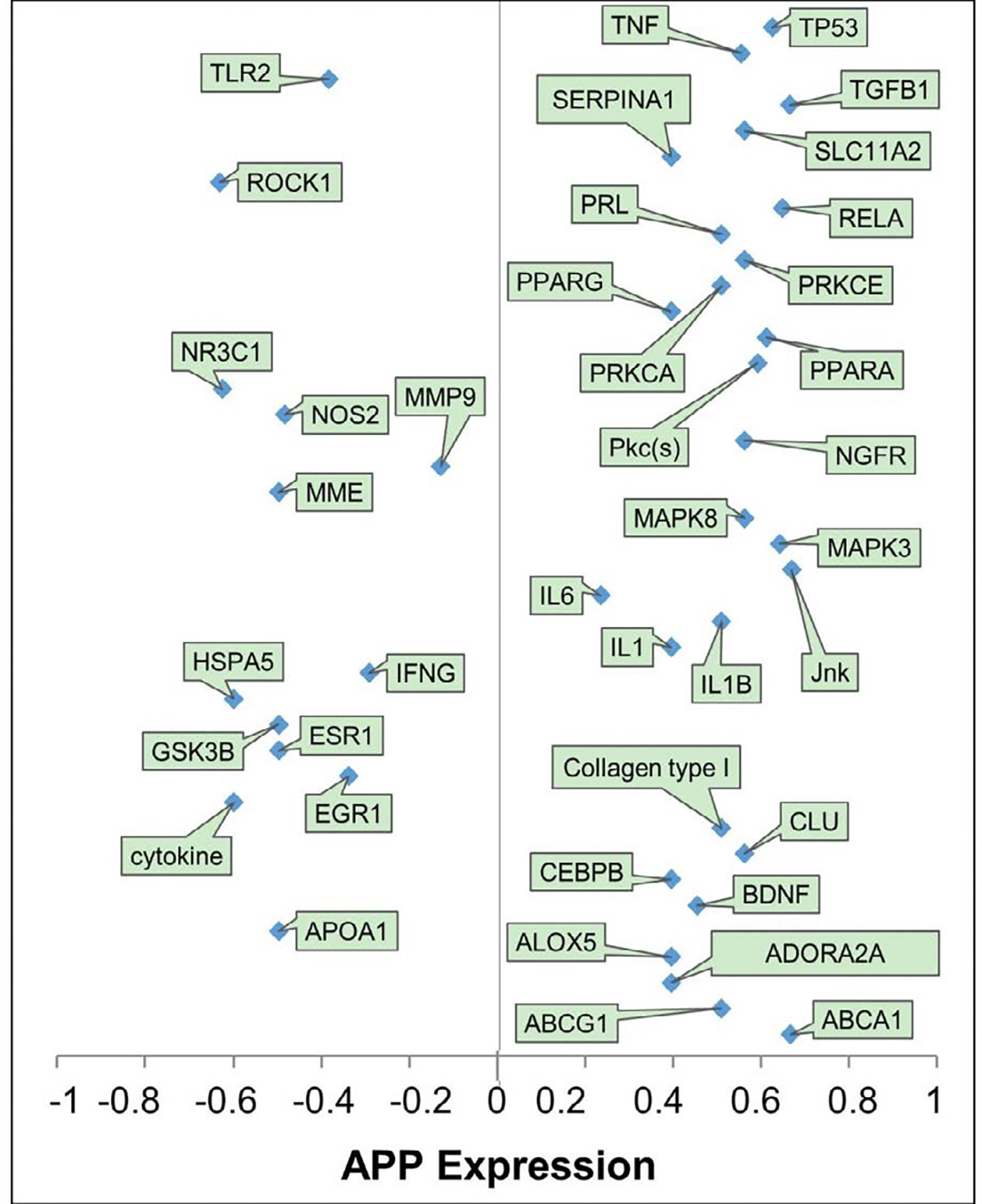
Quantitative illustration of the involvement of the 40 intermediary molecules in exposure to EtOH-mediated expression of APP showing the contributions of the individual molecule changes to the overall conclusion on the change in APP expression in response to EtOH exposure.

IPA’s algorithmic z-score was used to identify EtOH-induced gene expression changes that are associated with increasing or decreasing effects on APP expression. Of the 40 molecules with observed gene expression change in response to EtOH exposure, 13 molecules were predicted to decrease APP expression and 27 molecules were predicted to increase APP expression (Figure 3). The weights assigned to the 40 molecules are aggregated in Figure 4. The weights indicate the direction of the change in APP expression, from −1 (decrease) to +1 (increase), and the confidence of that finding [41].

### 3.4 Connectivity Pathway Analysis of EtOH Metabolite Acetaldehyde and APP

**Figure 5:**
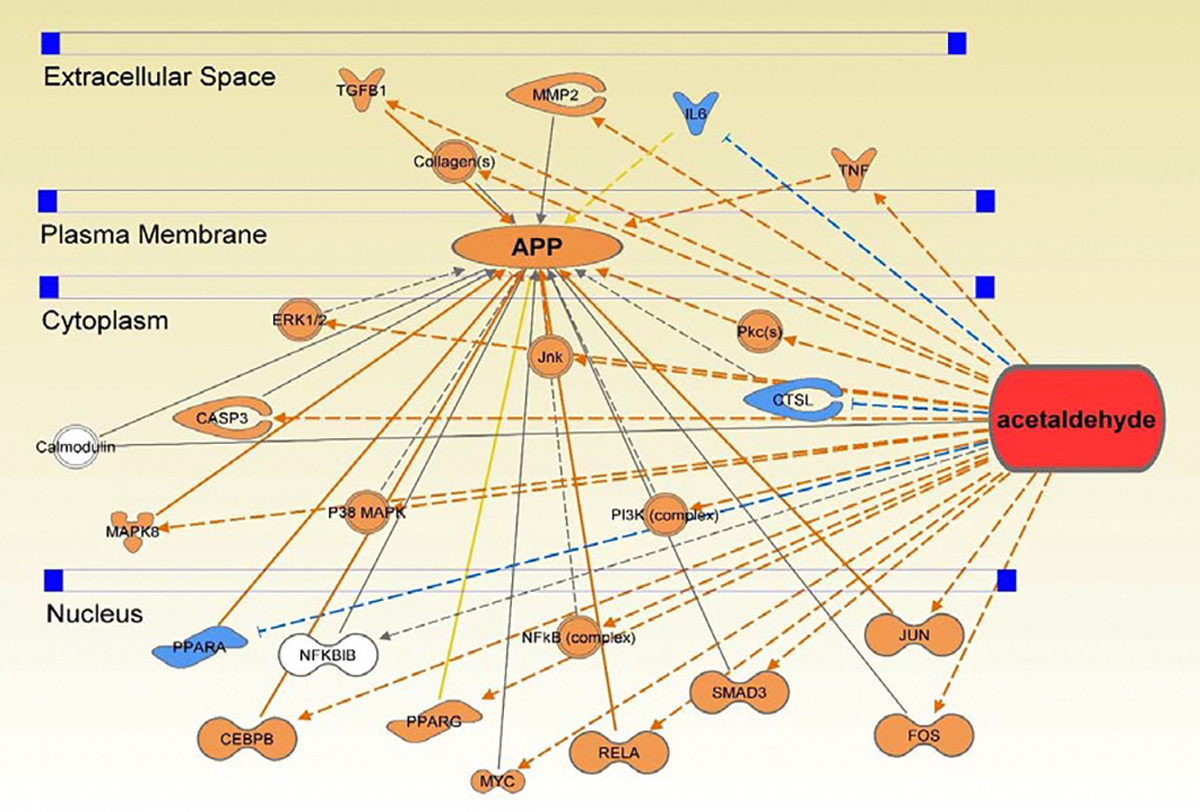
Connectivity Map of the direct (solid lines) and indirect (dotted lines) relationships between the 24 molecules identified that are associated with both acetaldehyde and APP. This suggests that the acetaldehyde metabolite of EtOH will lead to increased APP expression.

Using the “Grow” and “Pathway Explorer” tools of the “My Pathway” feature, 24 molecules associated with both acetaldehyde and APP were identified and organized into a connectivity map. The MAP tool was then used to reveal the influence of acetaldehyde on the 24 common molecules leading to a predicted increase in APP expression (Figure 5). A comparison of these 24 molecules with the 40 identified molecules from the analysis of EtOH exposure on APP revealed 11 common molecules. The presence of the 11 common molecules (Table 1)) suggest that a portion of the influence of EtOH on APP expression is mediated by the EtOH metabolite acetaldehyde; however, the remaining 29 molecules suggest that a holistic view of the influence of EtOH exposure on APP consists of other biological pathway cascades in addition to those mediated by acetaldehyde.

The pattern of expression changes observed in these 40 molecules is predicted to constitute a causal molecule network that results in a directional change in APP expression downstream. The statistical likelihood that the observed expression changes is consistent with what is known from the literature was evaluated by using the “Downstream Effect Analysis” algorithm as described by Krämer in 2014 [41]. Downstream effect analysis uses empirically based prior knowledge from the curated scientific literature, to establish the expected activation in the predicted causal molecule network relationships. In addition to consistency with the existing literature the analysis also calculates the direction of the expression change downstream. Thus, this analytical tool was used to test our hypotheses that the effect of EtOH exposure on the identified 40-molecule network leads to an increase in the expression of APP. The result revealed an increase in APP expression with the likelihood of finding an equally strong consistency by chance 5.61% of the time (z = 1.91, *p* = 0.0561 of a two-tailed distribution).

### 3.4 Pathway Analysis of the Overlapping EtOH and APP Molecules

**Figure 6:**
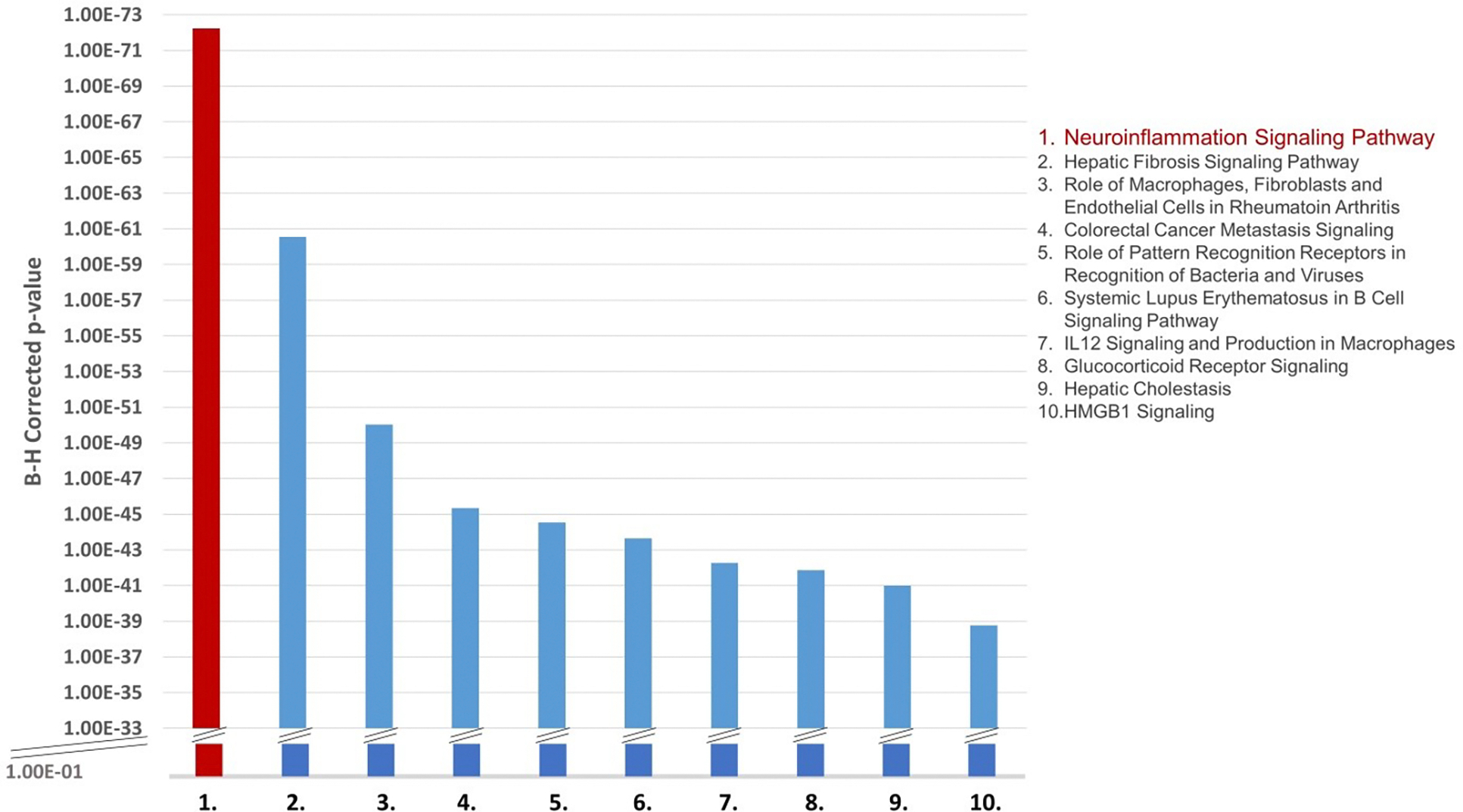
The top-ten canonical pathways ranked according to the overlap of the canonical pathway molecules and the 313 molecules associated with both EtOH and APP. For the list of the top ten identified canonical pathways with Benjamini-Hochberg corrected p-values, see Appendix – 2.

**Figure 7:**
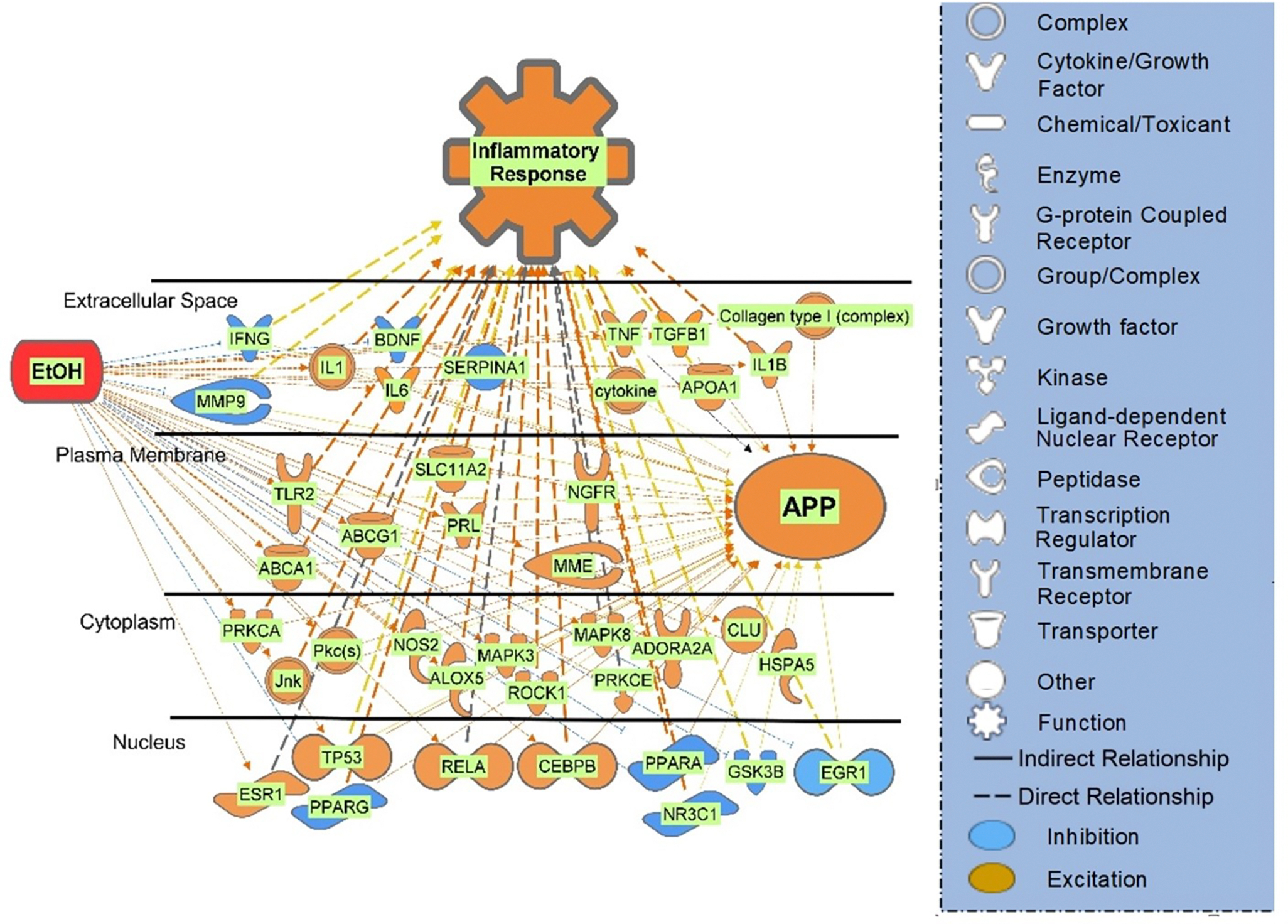
Connectivity Map of the direct (solid lines) and indirect (dotted lines) relationships of the 40 molecules which are influenced by EtOH exposure and which influence APP, of which, 31 molecules were found to be associated with inflammatory response functioning. For the full list of 40 molecules see Appendix – 1.

The next step was to determine if the identified molecules are associated with any of the biological pathways that are well characterized in the scientific literature (canonical pathways). The 313 molecules identified as being related to EtOH and APP were compared with the molecules of the 705 canonical signaling and metabolic pathways stored in the QKB. The statistical significance of the overlap between the 313-molecule set and the molecules in the canonical pathways were evaluated using a Benjamini-Hochberg corrected, one-tailed Fisher Exact Test. The top 10 most significantly identified pathways are presented in Figure 6 and listed in Appendix 2A. The canonical pathway found to have the strongest overlap with the 313 molecules related to EtOH and APP was the neuroinflammation signaling pathway (22.9% overlap, *p* = 5.97E-73). To determine if the 40 molecules that were identified to be impacted by EtOH and to influence APP are also associated with inflammatory response modulation, the “Grow” tool was used to grow the pathway for each molecule. A Connectivity Map of the 31 of the 40 molecules that were associated with inflammatory response modulation is shown in Figure 7.

Given the complexity and reactivity of the immune system it is possible that any canonical pathway analysis would result in an overlap of molecules from the neuroinflammation signaling pathway. A clear bias favoring the inclusion of neuroinflammation signaling in a canonical pathway analysis would weaken the support of the hypothesis that EtOH exposure effects on APP expression is mediated through neuroinflammation. To verify that no such biasing occurred, an analysis was run between APP and fertility, which has no a-priori associations. A canonical pathway analysis on 152 molecules associated with both APP and fertility revealed the most significant Benjamini-Hochberg corrected p-value to be *p* = 1.91E-10. Moreover, the neuroinflammation signaling pathway did not show up in the top ten identified pathways. Among the top ten canonical pathways that were identified (Appendix 2B), none matched the top ten identified in the APP and EtOH analysis. Thus, the result of this negative control increases confidence that neuroinflammation did not appear at the top of the associated pathway list because of broad and non-specific associations with many terms but because neuroinflammation directly mediates the relationship between EtOH exposure and APP expression.

## 4. Discussion

These data suggest that EtOH exposure increases the expression of APP. This may be the biological mechanism which has led to the finding that individuals who heavily drink alcoholic beverages may experience higher risk of the development of AD [2,42]. The finding of the present study is consistent with the view of AD as a polygenic disorder that is characteristic of late-onset sporadic AD. Although APOE4 allelic variation plays a role in the risk to AD other environmental factors also contribute to risk. Our findings suggest that alcohol consumption is an environmental factor that could lead to exacerbation of AD pathology, since increased Aβ deposition is the driving factor in this disease. This may also be true in familial AD pathology where there is overexpression of mutant APP and presenilin genes in early age, which may be exacerbated by EtOH exposure. Our pathway analysis suggests that inflammation associated with AD [43] caused by EtOH exposure leads to increased APP expression and worsening of AD pathology.

Chronic alcohol consumption has been shown to increase systemic inflammation by inducing intestinal hyperpermeability (leaky gut). Alcohol induces hyperpermeability through the disruption of epithelial cells leading to increased transmission of pathogens and toxic substances into the bloodstream. This leads to increased endotoxin concentrations (i.e. LPS) and associated systemic inflammation [31–36]. The toxicity caused by EtOH has been hypothesized to be the cause of alcohol-associated organ damage, and a major contributing factor to liver disease [44]. Liver injury may contribute to systemic inflammation and neuroinflammation leading to AD by damaging the cholinergic anti-inflammatory arm of the inflammatory reflex [37,38]. The present study reveals the role of neuroinflammation in the mediation of APP expression and AD pathology following EtOH exposure.

**Figure 8:**
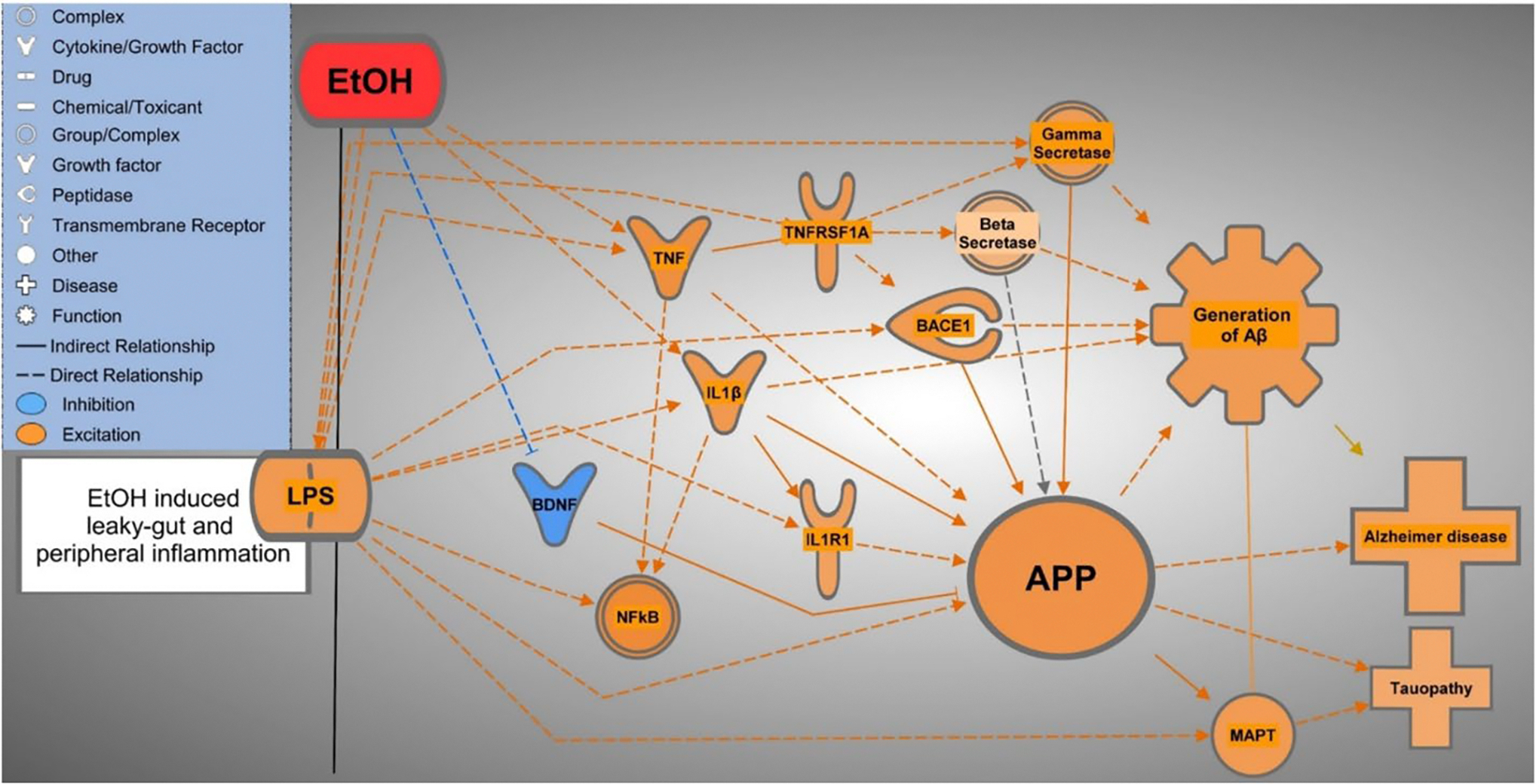
Molecule network illustrating the direct (solid lines) and indirect (dotted lines) neuroinflammatory molecule relationships between EtOH exposure, APP expression, Aβ secretion, and related functions and diseases. This illustration was produced using the Pathway Designer tool of IPA.

Examining the role of the 31 molecules which were influenced by EtOH exposure and which influence APP in the neuroinflammation signaling pathway revealed the influence of EtOH on well-known neuroinflammatory molecules, APP expression, and Aβ secretion (Figure 8). The increase in LPS levels from intestinal hyperpermeability corroborates the increased expression of Toll-Like Receptor 2 (TLR2), one of the 31 molecules influenced by EtOH and that influence APP, that was found in the neuroinflammation signaling pathway. TLR2 has been shown to be regulated by LPS and involved in Aβ binding [45–48].

Another of the 31 molecules found in the neuroinflammation signaling pathway, brain-derived neurotrophic factor (BDNF) was shown to decrease expression following EtOH exposure [49]. BDNF is an important member of the nerve growth factor family, responsible for neuronal survival. The down regulation of BDNF, as occurs in response to EtOH exposure, is associated with the worsening of AD pathology and increased APP expression [50]. This suggests that the impact of EtOH on BDNF contributes to the increased risk of AD pathology following alcohol consumption, through decreased neuronal survival and increased APP expression.

EtOH exposure was shown to modulate neurotrophic factor kappa-B (NFkB)-mediated neuroinflammation and associated pro-inflammatory proteins. Specifically, EtOH increases the expression of tumor necrosis factor alpha (TNFα) and interleukin 1 beta (IL-1β) [51]. Increased TNFα expression has been shown to increase the expression of TNFRSF1A [52], associated with increased activity of the γ- and β-secretase complexes, which are responsible for the pathological cleaving of APP into Aβ. Future studies may investigate the impact of EtOH exposure on the secretase complexes as another potential mediating biological system between EtOH and AD pathology [53].

One of the 9 non-inflammation associated molecules from the 40 that were shown to change APP expression and respond to EtOH exposure is solute carrier family 11 member 2 (SLC11A2). SLC11A2 and other similar solute carriers found in the 313 overlapping molecules between EtOH and APP (solute carrier family 2 member 2 and solute carrier family 40 member 1) show increased expression following EtOH exposure leading to increased transmigration of amino acids and minerals (iron, copper, zinc) and the disruption of the iron homeostasis signaling [54,55]. Disruption of mineral homeostasis has been associated with increased APP expression [56–60].

The increased expression of APP caused by EtOH exposure has troubling implications beyond AD pathology. Increased expression of APP has been associated with expression of microtubule associated protein tau (MAPT), which leads to formation of “tau tangles” [61,62]. Tau tangles are a component of several neurodegenerative diseases, including AD, collectively referred to as “Tauopathies.” These findings suggest that EtOH exposure may also lead to increased Tau tangle formation and detrimental effects in associated neurodegenerative disorders including Parkinson’s and Huntington’s Disease [63].

The relevance of the acetaldehyde component of the influence of EtOH on APP is made more apparent when considering the canonical pathways that were identified through the canonical pathway analysis of the 313 molecules associated with both EtOH and APP through which the Hepatic Fibrosis Signaling Pathway (*p* = 2.85E-61) and Hepatic Cholestasis Pathway (1.00E-41) were revealed to have the second and ninth most significant overlap p-values. Since the toxic metabolite acetaldehyde is believed to play a major role in the liver disease [64] these findings suggest that there are similar mechanisms between the role of alcohol consumption in APP expression and the role of alcohol consumption in liver disease and that epidemiological studies may reveal a positive correlation between liver disease and those who develop AD. However, given that over half of the molecules that were identified with EtOH and APP were not associated with the influence of acetaldehyde, a holistic picture of the influence of EtOH on APP suggests alternative mechanisms of EtOH are in part responsible for the role of alcohol consumption in APP expression. This is in line with the findings that EtOH has been shown to have low-affinity binding proteins [65], which would explain the need for higher concentrations to cause the deleterious impact of EtOH on risk of AD development [39,65,66].

The findings from our *in silico* studies have provided valuable information to develop therapeutic strategies against EtOH-induced AD risk. Involvement of inflammatory responses in EtOH modulation of APP expression, as we have identified, has suggested that some anti-inflammatory compounds might be beneficial to decrease AD risk of alcoholic patients. In particular, the anti-inflammatory, anti-Aβ compound, *Withaferin A* has been shown to destabilize Aβ fibrils and reduce neuroinflammation, which according to our findings, would provide a therapeutic, protective benefit against AD development [67,68]). This research also supports the hypothesis that the anti-inflammatory properties of docosahexaenoic acid may contribute to its neuroprotective role in animal models of AD [69,70]. In addition, management of alcohol use-induced intestinal hyperpermeability (leaky gut) to reduce peripheral inflammation would be necessary, along with any treatments to reduce the buildup of Aβ secretions, would be most effective to reduce risk of AD caused by heavy alcohol consumption.

## Supplementary Material

Appendix

## Figures and Tables

**Table 1:** 40 Molecules that are affected by EtOH and Influence APP

Abb.	Entrez Gene Name
**ABCA1**	ATP binding cassette subfamily A member 1
**ABCG1**	ATP binding cassette subfamily G member 1
**ADORA2A** *	adenosine A2a receptor
**ALOX5** *	arachidonate 5-lipoxygenase
**APOA1** *	apolipoprotein A1
**BDNF** *	brain derived neurotrophic factor
**CEBPB** *	CCAAT enhancer binding protein beta
**CLU**	clusterin
**Collagen type I (complex)**	--
**Cytokine** *	--
**EGR1** *	early growth response 1
**ESR1** *	estrogen receptor 1
**GSK3B** *	glycogen synthase kinase 3 beta
**HSPA5**	heat shock protein family A (Hsp70) member 5
**IFNG** *	interferon gamma
**IL1** *	--
**IL1B** *	interleukin 1 beta
**IL6** *	interleukin 6
**Jnk** *	--
**MAPK3** *	mitogen-activated protein kinase 3
**MAPK8** *	mitogen-activated protein kinase 8
**MME**	membrane metalloendopeptidase
**MMP9** *	matrix metallopeptidase 9
**NGFR**	nerve growth factor receptor
**NOS2** *	nitric oxide synthase 2
**NR3C1** *	nuclear receptor subfamily 3 group C member 1
**Pkc(s)** *	--
**PPARA** *	peroxisome proliferator activated receptor alpha
**PPARG** *	peroxisome proliferator activated receptor gamma
**PRKCA** *	protein kinase C alpha
**PRKCE** *	protein kinase C epsilon
**PRL**	prolactin
**RELA** *	RELA proto-oncogene, NF-kB subunit
**ROCK1** *	Rho associated coiled-coil containing protein kinase 1
**SERPINA1** *	serpin family A member 1
**SLC11A2**	solute carrier family 11 member 2
**TGFB1** *	transforming growth factor beta 1
**TLR2** *	toll like receptor 2
**TNF** *	tumor necrosis factor
**TP53** *	tumor protein p53

* Indicates Connection with the Inflammation Response Function
