# Supplementary material for: Alcohol Modulation of Amyloid Precursor Protein in Alzheimer’s Disease
Source: J Drug Alcohol Res. Author manuscript; Available in PMC 2026 Mar 21. (PMC13004628)
Supplement: Appendix [file NIHMS2108481-supplement-Appendix.pdf]

Appendix – 1: 40 Molecules that are affected by EtOH and Influence APP

Abb.	Entrez Gene Name
ABCA1	ATP binding cassette subfamily A member 1
ABCG1	ATP binding cassette subfamily G member 1
ADORA2A*	adenosine A2a receptor
ALOX5*	arachidonate 5-lipoxygenase
APOA1*	apolipoprotein A1
BDNF*	brain derived neurotrophic factor
CEBPB*	CCAAT enhancer binding protein beta
CLU	clusterin
Collagen type I (complex)	--
Cytokine*	--
EGR1*	early growth response 1
ESR1*	estrogen receptor 1
GSK3B*	glycogen synthase kinase 3 beta
HSPA5	heat shock protein family A (Hsp70) member 5
IFNG*	interferon gamma
IL1*	--
IL1B*	interleukin 1 beta
IL6*	interleukin 6
Jnk*	--
MAPK3*	mitogen-activated protein kinase 3
MAPK8*	mitogen-activated protein kinase 8
MME	membrane metalloendopeptidase
MMP9*	matrix metallopeptidase 9
NGFR	nerve growth factor receptor
NOS2*	nitric oxide synthase 2
NR3C1*	nuclear receptor subfamily 3 group C member 1
Pkc(s)*	--
PPARA*	peroxisome proliferator activated receptor alpha
PPARG*	peroxisome proliferator activated receptor gamma

PRKCA*	protein kinase C alpha
PRKCE*	protein kinase C epsilon
PRL	prolactin
RELA*	RELA proto-oncogene, NF-kB subunit
ROCK1*	Rho associated coiled-coil containing protein kinase 1
SERPINA1*	serpin family A member 1
SLC11A2	solute carrier family 11 member 2
TGFB1*	transforming growth factor beta 1
TLR2*	toll like receptor 2
TNF*	tumor necrosis factor
TP53*	tumor protein p53
* Indicates Connection with the Inflammation Response Function	

Appendix – 2A: Pathways associated with the 313 molecules identified between EtOH and APP

Canonical Pathway Name	Benjamini-Hochberg Corrected p-value
Neuroinflammation Signaling Pathway	5.97E-73
Hepatic Fibrosis Signaling Pathway	2.85E-61
Role of Macrophages, Fibroblasts and Endothelial Cells in Rheumatoid Arthritis	9.23E-51
Colorectal Cancer Metastasis Signaling	4.53E-46
Role of Pattern Recognition Receptors in Recognition of Bacteria and Viruses	2.97E-45
Systemic Lupus Erythematosus in B Cell Signaling Pathway	2.29E-44
IL12 Signaling and Production in Macrophages	5.32E-43
Glucocorticoid Receptor Signaling	1.35E-42
Hepatic Cholestasis	1.00E-41
HMGB1 Signaling	1.72E-39

Appendix – 2B: Top 10 pathways of the negative control analysis associated with the 152 molecules identified between APP and fertility.

Canonical Pathway Names	Benjamini-Hochberg Corrected p-value
Cardiac Hypertrophy Signaling (Enhanced)b	1.91E-10
Aryl Hydrocarbon Receptor Signaling	1.66E-09
Estrogen-mediated S-phase Entry	2.24E-08
Estrogen Receptor Signaling	2.51E-08
Molecular Mechanisms of Cancer	3.47E-08
Colorectal Cancer Metastasis Signaling	4.90E-08
Role of Macrophages, Fibroblasts and Endothelial Cells in Rheumatoid Arthritis	6.92E-08
Systemic Lupus Erythematosus In B Cell Signaling Pathway	1.05E-07
Glucocorticoid Receptor Signaling	1.45E-07
Osteoarthritis Pathway	4.27E-07
